# Influence of Magnetic Nanoparticles on Modified Polypyrrole/*m*-Phenylediamine for Adsorption of Cr(VI) from Aqueous Solution

**DOI:** 10.3390/polym12030679

**Published:** 2020-03-19

**Authors:** Thabiso Carol Maponya, Kabelo Edmond Ramohlola, Nazia Hassan Kera, Kwena Desmond Modibane, Arjun Maity, Lebogang Maureen Katata-Seru, Mpitloane Joseph Hato

**Affiliations:** 1 Nanotechnology Research Lab, Department of Chemistry, School of Physical and Mineral Sciences, University of Limpopo (Turfloop), Sovenga 0727, Polokwane, South Africa; 2209thabiso@gmail.com (T.C.M.); kabelo.ramohlola@ul.ac.za (K.E.R.); 2 DST/CSIR Innovation Centre, National Centre for Nanostructured Materials, CSIR Material Science and Manufacturing, Pretoria 0001, South Africa; Kera.Nazia@csir.co.za (N.H.K.); AMaity@csir.co.za (A.M.); 3 Chemistry Department, Faculty of Natural and Agricultural Sciences, North-West University, Mmabatho 2735, South Africa; Lebo.Seru@nwu.ac.za; 4 Department of Environmental Sciences, College of Agriculture and Environmental Sciences, University of South Africa (UNISA), Florida Science Campus, Johannesburg 1710, South Africa

**Keywords:** water pollution, *m*-phenylenediamine, magnetic polymer nanocomposite, kinetics, adsorption properties

## Abstract

A novel, modified polypyrrole/*m*-phenylediamine (PPy–*m*PD) composite, decorated with magnetite (Fe_3_O_4_) nanoparticles, and prepared via an in-situ oxidative polymerisation, was investigated. The PPy–*m*PD/Fe_3_O_4_ nanocomposite was employed for the removal of highly toxic oxyanion hexavalent chromium Cr(VI) from an aqueous solution. The structure and successful formation of the PPy–*m*PD/Fe_3_O_4_ nanocomposite was confirmed and investigated using various techniques. The presence of Fe_3_O_4_ was confirmed by high resolution transmission electron microscopy, with an appearance of Fe lattice fringes. The estimation of the saturation magnetisation of the nanocomposite, using a vibrating sample magnetometer, was observed to be 6.6 emu/g. In batch adsorption experiments, PPy–*m*PD/Fe_3_O_4_ nanocomposite (25 mg) was able to remove 99.6% of 100 mg/L of Cr(VI) at pH 2 and 25 °C. Adsorption isotherms were investigated at different Cr(VI) concentration (100–600 mg/L) and temperature (15–45 °C). It was deduced that adsorption follows the Langmuir model, with a maximum adsorption capacity of 555.6 mg/g for Cr(VI) removal. Furthermore, isotherm data were used to calculate thermodynamic values for Gibbs free energy, enthalpy change and entropy change, which indicated that Cr(VI) adsorption was spontaneous and endothermic in nature. Adsorption–desorption experiments revealed that the nanocomposite was usable for two consecutive cycles with no significant loss of adsorption capacity. This research demonstrates the application potential for the fascinating properties of PPy–*m*PD/Fe_3_O_4_ nanocomposite as a highly efficient adsorbent for the removal of heavy metal ions from industrial wastewater.

## 1. Introduction

Water is the life artery of living systems that is essential to human health and welfare, and a prerequisite to industrial development. Millions of people in the world are estimated to die each year as a result of unsafe drinking water [1]. For this reason, wastewater treatment is essential to the safety of human beings. The removal of toxic heavy metals, such as lead (Pb), cadmium (Cd), chromium (Cr) and copper (Cu), is one of the main problems for wastewater treatment [2].

These pollutants can enter water systems through industrial mining, galvanoplastic foundries, and from pipe corrosion. Therefore, a long-term exposure to these heavy metals can accumulate in living organisms, causing several disorders and serious diseases [2]. Thus, it is important to search for economical and efficient methods to protect water resources from pollution.

Polypyrrole (PPy) is a conducting polymer that has been studied to remove hexavalent chromium from water, due to its fascinating properties, such as its ease of chemical substitution, high chemical stability, ion exchange behaviour, and the ability to reduce toxic Cr(VI) to less toxic Cr(III) [1,2,3,4,5]. A number of studies have reported on the functionalisation of PPy to increase the number of amine functional groups, which can facilitate chelation or adsorption of Cr(VI) ions by serving as adsorption sites for Cr(VI) [4,6,7]. For example, polyacrylonitrile/polypyrrole (PAN/PPy) core–shell-structured nanofibers synthesised via the electrospinning technique for the removal of Cr(VI) with an adsorption capacity of 61.80 mg/g, was reported [6]. Kera et al. [2], synthesised a PPy/2,5-diaminobenzene sulfonic acid (DABSA) composite via an in-situ oxidative polymerisation of PPy in the presence of DABSA. The PPy/DABSA composite had an adsorption capacity of 303 mg/g at 25 °C. In another study, PPy-*m-*phenylenediamine (*m*PD) with a high adsorption capacity of 526 mg/g for Cr(VI) at 25 °C and initial pH 2, was reported [2]. The incorporation of *m*PD resulted in an increase in the number of amine functional groups and active sites for Cr(VI) uptake, and significantly enhanced the surface area of PPy. In all these studies, it has been realised that the application of PPy-based composite adsorbents for water treatment is challenging, owing to difficulties in separating these adsorbents from the aqueous media after an adsorption process [7,8,9]. Therefore, the magnetic separation process has been one of the most employed techniques to circumvent the separation problem of adsorbents from aqueous solutions [10]. Magnetic nanoparticles, such as maghemite (*γ*-Fe_2_O_3_), magnetite (Fe_3_O_4_) [11,12,13] and Jacobsite (MnFe_2_O_4_) [14,15], have received much attention to assist during the magnetic separation process, due to their large surface areas and good water dispersion ability [16]. In addition, these nanoparticles are advantageous for the adsorption process, because of their high surface to volume ratio, resulting in a higher adsorption capacity for metal removal compared to other materials [2,10,16].

A number of studies have been carried out using magnetic PPy-based nanocomposites for the adsorption of Cr(VI) from wastewater [2,10,17]. PPy decorated with reduced graphene oxide (rGO)-Fe_3_O_4_ composite with an adsorption capacity of 293.3 mg/g was reported [18]. PPy/Fe_3_O_4_ and PPy-PANI/Fe_3_O_4_ nanocomposites were synthesised for the removal of Cr(VI) by various researchers [2,8]. The authors found that the PPy/Fe_3_O_4_ and PPy-PANI/Fe_3_O_4_ nanocomposites showed adsorption capacities of 196.4 and 303 mg/g for Cr(VI) removal at pH 2 and 25 *°*C, respectively.

The aim of this study was to synthesise a highly effective PPy–*m*PD/Fe_3_O_4_ nanocomposite adsorbent by a simple in-situ oxidative polymerisation route for the adsorption of Cr(VI) from water. It is reported that the presence of *m*PD in the nanocomposite enhanced the adsorption capacity of the PPy [3]. Herein, Fe_3_O_4_ nanoparticles were incorporated into the PPy–*m*PD composite to enhance its Cr(VI) adsorption capacity, and to simplify the separation of the adsorbent from the aqueous solution after the adsorption process. The synthesised PPy–*m*PD/Fe_3_O_4_ nanocomposite was studied as an adsorbent for the removal of Cr(VI) from wastewater in batch-type experiments.

## 2. Experimental

### 2.1. Materials

Pyrrole monomer (Py) (98%), iron chloride (FeCl_3_), magnetite (Fe_3_O_4_) nanoparticles, *m*–phenylenediamine (*m*PD) and 1,5-diphenylcarbazide were purchased from Sigma-Aldrich (South Africa). Potassium dichromate (K_2_Cr_2_O_7_) was obtained from Minema Chemicals (Johannesburg, South Africa). Cr(VI) stock solution (1000 mg/L) was prepared by dissolving a pre-determined amount of K_2_Cr_2_O_7_ in deionised water. Py was distilled under vacuum for purification and stored in the refrigerator until used. Other chemicals used in this study were of analytical grade, and were used as received.

### 2.2. Synthesis of PPy–mPD/Fe_3_O_4_ Nanocomposite

PPy–*m*PD/Fe_3_O_4_ nanocomposite was prepared via the in-situ polymerisation of pyrrole and *m*PD monomers in the presence of Fe_3_O_4_, according to the method reported by Kera et al. [3]. Typically, Fe_3_O_4_ (0.05 g) was uniformly dispersed in 100 mL of deionised water by ultrasonication for 30 min. The magnetite solution was allowed to cool down to room temperature, and 0.8 g of *m*PD was added to the mixture and dissolved by shaking. Py monomer, 0.8 mL, was then transferred into the mixture, which was agitated to disperse the Py uniformly. FeCl_3_ solution was prepared in a separate container by dissolving 6 g of FeCl_3_ in 100 mL of deionised water. The FeCl_3_ solution was then transferred into the mixture all at once, and agitated by shaking for 10 min at room temperature. The reaction mixture was left for 24 h for the polymerisation process to take place. The black resultant precipitate was obtained from the reaction mixture through filtration and was washed with deionised water and acetone to obtain a colourless filtrate. Then, the product was dried at 60 *°*C for 24 h under vacuum (mass obtained = 1.12 g).

### 2.3. Characterisations

The elemental analysis, morphology, and mapping of the PPy homopolymer and PPy–*m*PD/Fe_3_O_4_ nanocomposite before and after Cr(VI) adsorption were studied by using field-emission scanning electron microscopy (FE-SEM) (Auriga Cobra focused-ion beam FIB-SEM, Carl Zeiss, Jena, Germany), high resolution transmission electron microscopy (HR-TEM) (JEOL-JEM 2100 microscope, Tokyo, Japan) operated at 200 keV, and coupled with an electron diffraction X-ray scattering (EDX) analyser. The structure of the PPy homopolymer and PPy–*m*DP/Fe_3_O_4_ nanocomposite before and after adsorption was confirmed using Attenuated Total Reflectance Fourier transform infrared with a germanium crystal-attenuated total reflectance Fourier transform infrared microscopy (ATR-FTIR) PerkinElmer Spectrum 100 spectrometer (PerkinElmer, Johannesburg, South Africa). For IR measurements, the frequency range, resolution and number of scans were in the range of 650–4000 cm^−1^, 4 cm^−1^ and 10 cm^−1^, respectively. X-ray diffraction (XRD) patterns of the PPy homopolymer and PPy–*m*PD/Fe_3_O_4_ nanocomposite before and after the removal of Cr(VI) were obtained using a PANalyticalX’Pert PRO-diffractometer (PANalytical, Eindhoven, The Netherlands). The spectra were achieved using the CuK_α_ radiation (λ = 1.5406 Å) at 45 kV/40 mA and 2*θ* values ranging between 5 and 90°. The Brunauer–Emmet–Teller (BET) analysis was performed at a low-temperature N_2_ adsorption–desorption technique, using a Micromeritics ASAP 2020 gas adsorption apparatus (Micromeritics, Norcross, USA). The thermal stability of the prepared nanocomposite was studied using a thermogravimetric analyser (TGA) Q500 (TA Instruments, New Castle, USA). Furthermore, the sample was heated from 25 to 800 °C at a heating rate of 10 °C/min and the flow rate of 50 mL/min in air. The magnetic properties of the PPy–*m*PD/Fe_3_O_4_ nanocomposite were studied using a vibrating sample magnetometer (VSM, Lakeshore, Westerville, USA) at room temperature and a maximum applied magnetic field of 9.6 kOe. Ion chromatography coupled to inductively coupled plasma mass spectroscopy (IC-ICP-MS) (ThermoFisher Scientific instrumentation, Waltham, USA) was utilised to determine the concentration of Cr(VI) and Cr(III) ions present in the solutions obtained from the effect of the pH experiment. The measurement of Cr(VI) concentrations in solutions after adsorption by the PPy–*m*PD/Fe_3_O_4_ nanocomposite was conducted using an ultraviolet-visible (UV-vis) spectrophotometer, (Perkin Elmer, Johannesburg, South Africa).

### 2.4. Batch Adsorption Experiments

Batch adsorption experiments were executed to study the effect of adsorbent dose, solution pH, initial Cr(VI) concentration, temperature and co-existing ions on Cr(VI) adsorption by PPy–*m*PD/Fe_3_O_4_ nanocomposite. Different concentrations of Cr(VI) solution were prepared by diluting the 1000 mg/L Cr(VI) stock solution to evaluate these various parameters. For adsorption equilibrium experiments, a fixed amount of the nanocomposite (0.025 g) was contacted with 50 mL of 100 mg/L Cr(VI) solution in 100 mL plastic bottles in a thermostat shaker agitated for 24 h at a speed of 200 rpm. For pH studies, the pH of Cr(VI) solution was varied from 2.0–11.0 adjusted with HCl and NaOH solutions with 100 mg/L initial Cr(VI) concentration. 

The point of zero charge (pH_pzc_) of the PPy-*m*PD/Fe_3_O_4_ nanocomposite was determined by the pH drift method. To a series of 100 mL plastic bottles, 50 mL of 100 mg/L Cr(VI) was added, and the initial pH (pHi) was adjusted from 2–11 with 0.1 M HCl or 0.1 M NaOH solutions. To the adjusted pH solutions, 25 mg of PPy-*m*PD/Fe_3_O_4_ nanocomposite was added and the suspensions were placed in a thermostat shaker agitated for 24 h at a speed of 200 rpm. After 24 h, the suspensions were separated by filtration, and the final pH values of supernatant liquid were recorded.

The effect of adsorbent dose was carried out by varying the mass of the PPy–*m*PD/Fe_3_O_4_ nanocomposite from 0.005–0.05 g. Adsorption isotherms experiments were conducted at 15, 25, 35 and 45 °C to study Cr(VI) removal by the nanocomposite, while varying the concentration of the Cr(VI) from 100 to 600 mg/L.

For adsorption kinetics experiments, solutions with four initial Cr(VI) concentrations (25, 50, 75 and 100 mg/L) were prepared and contacted with PPy–*m*PD/Fe_3_O_4_ adsorbent to examine the contact time effect on the removal of Cr(VI). In a typical experiment, the PPy–*m*PD/Fe_3_O_4_ nanocomposite (0.5 g) was mixed with 1 L of 100 mg/L Cr(VI) solution at a pH of 2 in a temperature-controlled water bath and an overhead stirrer. The mixture was stirred at 200 rpm with a constant temperature of 25 °C, and a few aliquots (7.0 mL) were taken at selected time intervals. The collected samples were filtered using 0.45 μm cellulose acetate syringe filters, and then they were analysed.

Different ions (Cu^2+^, Zn^2+^, Co^2+^, Ni^2+^, NO_3_^−^, PO_4_^3−^, SO_4_^2−^ and Cl^−^) were selected to examine the effect of co-existing ions at different concentrations ranging from 20–100 mg/L in solutions on Cr(VI) removal by the PPy–*m*PD/Fe_3_O_4_ nanocomposite. The removal efficiency and the behaviour of the nanocomposite was obtained with the help of the following expressions (Equations (1) and (2)):

(1) $$\%\;\mathrm{removal}=\left(\frac{C_{o}-C_{e}}{C_{o}}\right)X\;100$$

(2) $$q_{e}=\left(\frac{C_{o}-C_{e}}{m}\right)\;V$$

where *C_o_* and *C_e_* represent the initial and equilibrium concentrations of Cr(VI) in mg/L, respectively. *q_e_* denotes the quantity of Cr(VI) adsorbed at equilibrium/unit mass of PPy–*m*PD/Fe_3_O_4_ adsorbent (mg/g). Both where *m* (g) and *V* (L) represent the mass of the adsorbent and the volume of Cr(VI) solution used, respectively.

Nonlinear and linear forms of the Langmuir and Freundlich isotherm models are presented in the following expressions, respectively, (Equations (3)–(6)) [3].

(3) $$\frac{q_{e}}{q_{m}}=\frac{K_{L}C_{e}}{1+K_{L}C_{e}}$$

(4) $$q_{e}=K_{F}\;C_{e}^{1/n}$$

(5) $$\frac{Ce}{qe}=\frac{1}{qmK_{L}}+\frac{Ce}{qm}$$

(6) $$\ln qe=lnK_{F}+\frac{1}{n}lnC_{e}$$

where *q_m_* (mg/g), *K_L_* (L/mg), *K_F_* (mg/g) and *n* each represent the Langmuir maximum adsorption capacity, Langmuir constant, Freundlich constant and adsorption intensity, respectively [2,3].

The thermodynamic parameters, such as the Gibbs free energy change (Δ*G*^0^), enthalpy change (Δ*H*^0^) and entropy change (Δ*S*^0^) for the adsorption process can be determined from the relationship of temperature and adsorption isotherms. Particularly, Δ*G*^0^ can be calculated using Equation (7).

(7) $$\Delta G^{0}=-RTlnK_{c}$$

To determine the values of Δ*S*^0^ and Δ*H*^0^, a graph of ln *K_c_* against 1/*T* (Figure S1) was plotted according to the following expressions:

(8) $$\ln K_{c}=\frac{\Delta S^{0}}{R}-\frac{\Delta H^{0}}{RT}$$

(9) $$K_{c}=m\frac{q_{e}}{C_{e}}$$

where *K_c_* (L/mol) is the equilibrium constant which represents the ratio of equilibrium concentration of Cr(VI) adsorbed onto the PPy-*m*PD/Fe_3_O_4_ nanocomposite to that in the bulk solution, *R* (J/mol/K) is the ideal gas constant, *T* (K) is the temperature, and *m* (g/L) is the adsorbent dose.

To determine the adsorption rate and other adsorption kinetics parameters, the data obtained was fit to the nonlinear and linear forms of the pseudo-first-order (PFO) and the pseudo-second-order kinetic (PSO) models with the help of the following expressions:

(10) $$\frac{dq_{t}}{dt}=k_{1}\left(q_{e}-q_{t}\right)$$

(11) $$\frac{dq_{t}}{dt}=k_{2}{\left(q_{e}-q_{t}\right)}^{2}$$

(12) $$ln\left(q_{e}-q_{t}\right)=lnq_{e}-k_{1}t$$

(13) $$\frac{t}{q_{t}}=\frac{1}{k_{2}\;q_{t}^{2}}+\frac{1}{q_{e}}t$$

where *k*_1_ (1/min) represents pseudo-first-order rate (PFOR) constant and *k*_2_ (g/mg.min) denotes the pseudo-second-order rate (PSOR) constant.

## 3. Results and Discussion

### 3.1. Morphological Characterisations

**Figure 1:**
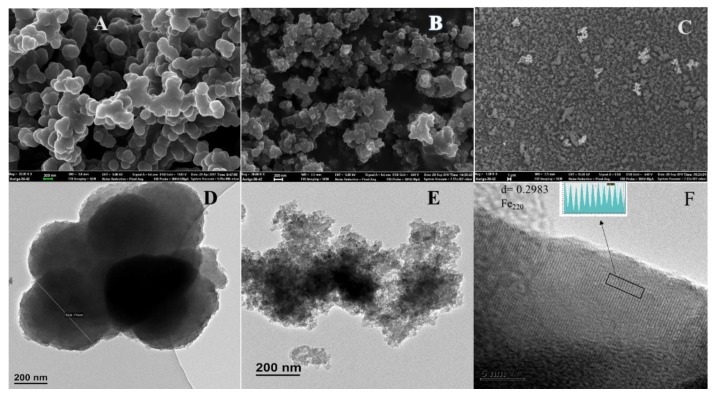
Scanning electron microscopy (SEM) images of polypyrrole (PPy) (**A**) and polypyrrole-*m*-phenylediamine/Fe_3_O_4_ (PPy–*m*PD/Fe_3_O_4_) nanocomposite (**B**,**C**); Transmission electron microscopy (TEM) images of PPy (**D)** and PPy–*m*PD/Fe_3_O_4_ nanocomposite (**E**); High resolution transmission electron microscopy (HR-TEM) of the PPy–*m*PD/Fe_3_O_4_ nanocomposite (**F**). N.B: Field-emission scanning electron microscopy (FE-SEM) image of the nanocomposite carried out in the backscattered mode (**C**).

**Figure 2:**
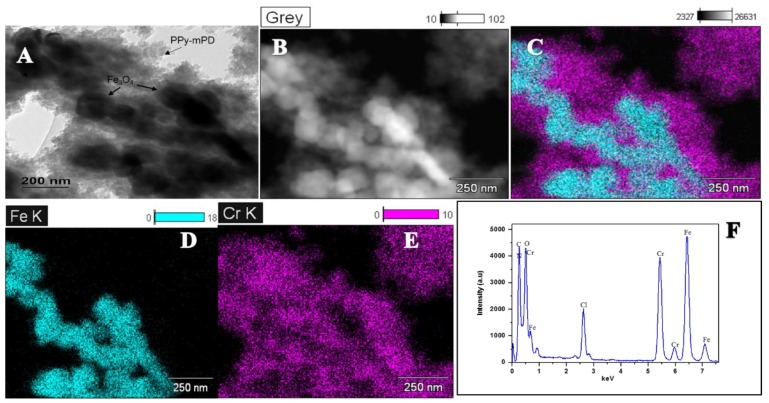
(**A**) HR-TEM images, (**B**–**E**) elemental mapping images, and electron diffraction X-ray scattering (EDX) spectra (**F**) of the PPy–*m*PD/Fe_3_O_4_ nanocomposite after Cr(VI) adsorption.

The morphology and structure of the PPy and its nanocomposite are shown in Figure 1. FE-SEM images are depicted in Figure 1a–c, TEM (Figure 1d,e) and the HR-TEM image in Figure 1f. Figure 1a–c illustrate the PPy and the nanocomposite. As seen in the FE-SEM and TEM images in Figure 1a,d, the PPy homopolymer comprises highly agglomerated spherical-shaped particles, which are similar to those reported by Chen and coworkers [19]. The particle size of the PPy–*m*PD/Fe_3_O_4_ nanocomposite depicted in Figure 1b,e are smaller than that of the PPy homopolymer. The presence and distribution of Fe_3_O_4_ nanoparticles in the funtionalised PPy with *m*PD was observed in the FE-SEM images obtained using the backscatter mode presented in Figure 1c. It can be noticed that the nanoparticles appear as bright spots with an observable agglomerated morphology. The TEM image in Figure 1f depicts lattice fringes possessing an interplanar d-spacing of 0.2983 nm. The obtained d-spacing of 0.2983 nm is assigned to the (220) plane of the cubic spinel structure of Fe_3_O_4_ nanoparticles [20]. Figure 2a–e present HR-TEM and elemental mapping images of the PPy–*m*PD/Fe_3_O_4_ nanocomposite after the adsorption of Cr(VI). From these images, it was noticed that the Cr ions are adsorbed onto the nanocomposite. This observation confirms the successful incorporation of Fe_3_O_4_ nanoparticles into the nanocomposite, and the adsorption of Cr(VI). The corresponding EDX spectrum depicted in Figure 2f shows peaks corresponding to C (0.3 keV), N (0.4 keV), O (0.5 keV), Cl (2.7 keV), Fe (6.5 keV and 7.2 keV) and Cr (5.5 keV and 6 keV).

### 3.2. Fourier Transform Infrared Analysis

**Figure 3:**
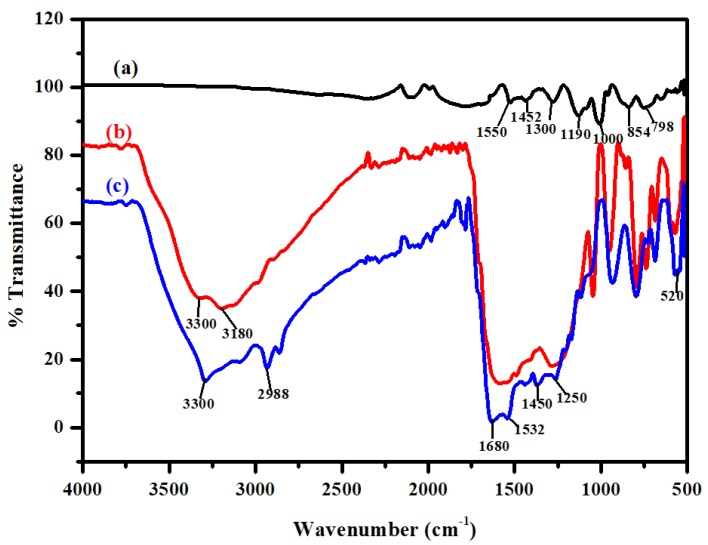
Fourier transform infrared (FTIR) spectra of PPy (**a**), PPy-*m*PD/Fe_3_O_4_ nanocomposite before (**b**) and after (**c**) Cr(VI) adsorption.

Fourier transform infrared (FTIR) spectra of PPy and PPy–*m*PD/Fe_3_O_4_ nanocomposite, before and after Cr(VI) adsorption, are provided in Figure 3. Figure 3a shows the bands obtained for the PPy homopolymer at 798, 854, 1000, 1190, 1300, 1452 and 1511 cm^−1^, corresponding to the C–H out-of-plane ring deformation of pyrrole, C–H in-plane deformation, N–H in-plane deformation, C–N stretching, C–N ring stretching, as well as the ring stretching mode of C=C [21]. The presence of Fe_3_O_4_ nanoparticles in the PPy–*m*PD/Fe_3_O_4_ nanocomposite before the adsorption of Cr(VI) is confirmed by the intense band at 520 cm^−1^ as shown in Figure 2b. The band at 3180 cm^−1^ is attributed to the N–H stretching vibration of the *m*PD in the nanocomposite. In addition, the vibration band at 3300 cm^−1^ is associated with the presence of moisture in the nanocomposite [22].

The FTIR spectrum of nanocomposite after Cr(VI) adsorption is given in Figure 2c. It is noticeable that there is a shift in the N–H vibration band appearing at 3180 cm^−1^ to 2988 cm^−1^. The shift is due to electrostatic interaction of HCrO_4_^−^ on the PPy–*m*PD/Fe_3_O_4_ nanocomposite [23]. Furthermore, the nanocomposite revealed bands at 1680, 1532 and 1250 cm^−1^, corresponding to the quinoid imines, benzoid amines and C–N stretching of *m*PD, respectively.

### 3.3. X-ray diffraction, Brunauer–Emmet–Teller, Thermogravimetric Analysis and Vibrating Sample Magnetometer Studies

**Figure 4:**
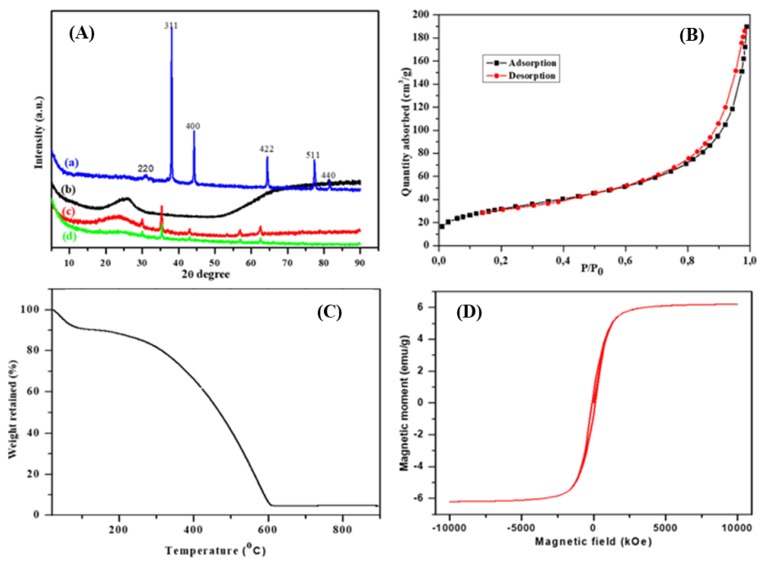
X-ray diffraction (XRD) patterns (**A**) of (**a**) Fe_3_O_4_ nanoparticles, (**b**) PPy and PPy–*m*PD/Fe_3_O_4_ nanocomposite before (**c**) and after (**d**) Cr(VI) adsorption. Brunauer–Emmet–Teller (BET) spectrum (**B**), thermogravimetric analysis (TGA) thermogram (**C**) and vibrating sample magnetometer (VSM) plot (**D**) of the PPy–*m*PD/Fe_3_O_4_ nanocomposite.

XRD, BET, TGA and VSM analyses were conducted to support the FTIR findings for the successful synthesis of the nanocomposite, and the results are presented in Figure 4. The XRD analysis was done, and the diffraction patterns of the synthesised PPy, magnetite nanoparticles and the PPy–*m*PD/Fe_3_O_4_ nanocomposite prior to and after Cr(VI) adsorption, are given in Figure 4A(a–d). The XRD pattern of the Fe_3_O_4_ nanoparticles (Figure 4A(a)) exhibits sharp peaks appearing at 2*θ* = 32.2, 38.0, 44.4, 64.7, 78.1 and 83.5°, assigned to the (220), (311), (400), (422), (511) and (440) planes, respectively, indicating the highly crystalline phase purity of the nanoparticles [20]. These results agree well with the HR-TEM results in Figure 1f. Figure 4A(b) shows the XRD pattern of the PPy with a broad peak appearing at 2*θ* = 25°, suggesting an amorphous structure of the PPy homopolymer [2]. It was seen that upon the nanocomposite formation (Figure 4A(c)), the patterns shifted to lower 2θ values as compared to PPy homopolymer and Fe_3_O_4_ nanoparticles with the disappearance of peak intensity of the 511 and 440 planes. Furthermore, Figure 4A(d) is the XRD pattern of the PPy-*m*PD/Fe_3_O_4_ nanocomposite after Cr(VI) adsorption, which shows the reduction in peak intensity at 2θ = 25°, indicating the presence of adsorbed Cr(VI) on the surface of the PPy-*m*PD/Fe_3_O_4_ nanocomposite [3,6].

The BET surface area of the PPy–*m*PD/Fe_3_O_4_ nanocomposite was obtained using the N_2_ adsorption/desorption curves, and the results are presented in Figure 4B. It is noticeable that the nanocomposite shows the type IV isotherm hysteresis loop, suggesting the mesoporous nature of the nanocomposite [23]. The BET specific surface area, average pore volume and diameter of the nanocomposite, were found to be 120.63 m^2^/g, 0.2961 m^3^/g and 11.69 nm, respectively. In another study [2], it was shown that the BET specific surface areas of the PPy and PPy-*m*PD nanocomposites were 5.3 and 183.2 m^2^/g, respectively. The authors concluded that an addition of mPD into the PPy increased the surface area, owing to a reduction in the particle size. In our study, a reduction in the surface area for the PPy–*m*PD/Fe_3_O_4_ nanocomposite may be due to nanoparticles entering or partially blocking some of the PPy-*m*PD nanocomposite pores [6].

The thermal stability of the PPy–*m*PD/Fe_3_O_4_ nanocomposite was studied using TGA, and the result is shown in Figure 4C. It can be seen that the nanocomposite shows the mass loss at 100 °C, due to the release of moisture, and the mass decrease between 100–200 °C, which is attributable to the disintegration of lower molecular weight oligomers that are present [3]. The degradation of the nanocomposite took place from 140–600 °C, implying that below 140 °C, the nanocomposite is thermally stable, and can be used for water purification processes performed at ambient conditions. The percentage weight retained of the Fe_3_O_4_ nanoparticles at the end of the cycle was about 5.0 wt %.

The VSM plot was used to study the magnetic behaviour of the synthesised PPy–*m*PD/Fe_3_O_4_ nanocomposite measured at 300 K, and the data are given in Figure 4D. The magnetic hysteresis loop exhibits that the saturation magnetisation (*M*_s_) value of the nanocomposite was 6.6 emu/g. This value is less than that reported in the literature for the Fe_3_O_4_/PPy nanocomposite, which might be due to the small amount of Fe_3_O_4_ nanoparticles used for the preparation of the nanocomposite [23]. Again, magnetisation hysteresis of the nanocomposite was close to zero, and this is common for superparamagnetic-based iron oxide nanoparticles, and confirms their superparamagnetic nature of the nanocomposite synthesised in this study [24]. Even though there was a reduction in *M*_s_ value, the nanocomposite still possesed enough magnetic power to be separated by an external magnetic field from aqueous solution after the removal of Cr(VI).

### 3.4. Adsoprtion Properties

To perform batch adsorption experiments, the effect of adsorbent dose, solution pH, initial Cr(VI) concentration, temperature, and the co-existing ions on Cr(VI) adsorption by PPy–*m*PD/Fe_3_O_4_ nanocomposite, were investigated.

**Figure 5:**
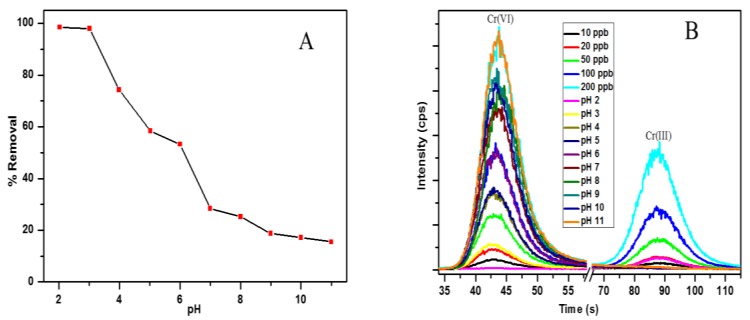
The effect of pH on Cr(VI) by adsorbent (**A**) and IC-ICP-MS chromatograms for analysis of chromium speciation (**B)** in standard solutions and the solutions obtained from the pH effect experiments.

The pH of the solution is the most vital parameter to be considered for the adsorption of heavy metal ions, because it affects the surface charge of the adsorbent, as well as the speciation of the metal ions in solution [25]. Figure 5a shows the effect of pH on the removal efficiency of Cr(VI) by the PPy–*m*PD/Fe_3_O_4_ nanocomposite. It can be seen that there is a reduction in the removal efficiency of Cr(VI) as the pH was increased from 2.0–11.0. To determine the surface charge of the adsorbent, the point of zero charge (pH_pzc_) was done. The pH_pzc_ is simply the pH at which the adsorbent surface has a net charge of zero. It is obtained from the difference between the initial pH_i_ and the final pH (pH_f_). The plot of ΔpH as a function of pHi is presented in the Supplementary Information (Figure S2) for the determination of pH_pzc_ for the PPy–*m*PD/Fe_3_O_4_ nanocomposite. From the results, it was observed that the nanocomposite has a pH_pzc_ at a pH of 2.3, which correlates well with the pH studies, since the maximum adsorption capacity was obtained at pH 2 and decreased with increasing pH. At pH up 2–6, the most prevailing Cr(VI) species in solution are the HCrO_4_^−^ and Cr_2_O_7_^2−^ oxyanions, which become CrO_4_^2−^ as the pH increases above 6 [26]. At pH 2, the nitrogen atoms of the amino groups (–NH_2_) on the surface of the nanocomposite are protonated (–NH_3_^+^), and attract the negatively-charged Cr(VI) oxyanions. This results in the adsorption of the Cr(VI) anions onto the surface of the PPy–*m*PD/Fe_3_O_4_ nanocomposite [26]. The decrease in the removal efficiency of Cr(VI) as the pH rises is due to an increased competition between the hydroxyl (OH^−^) ions and the CrO_4_^2−^ anions for the active adsorption sites (–NH_3_^+^) on the surface of the nanocomposite, and this results in more Coulombic repulsion anions [27]. Speciation studies were carried out using IC-ICP-MS to determine the concentrations of Cr(III) and Cr(VI) in solutions, and the results are given in Figure 5b. From the data given in Figure 3b, it is noticeable that at pH 2, Cr(III) species occur in the solution after Cr(VI) removal. This confirms that the PPy–*m*PD/Fe_3_O_4_ nanocomposite has the ability to both adsorb and reduce Cr(VI) to Cr(III). The HCrO_4_^−^ form which exists at pH 2 has a high redox potential, and is therefore reduced to Cr(III) by the nanocomposite [1,2], as shown in this expression (Equation (14)):

HCrO_4_^−^ + 7H^+^ + 3e^−^ ↔ Cr^3+^ + 4H_2_O (14)

**Figure 6:**
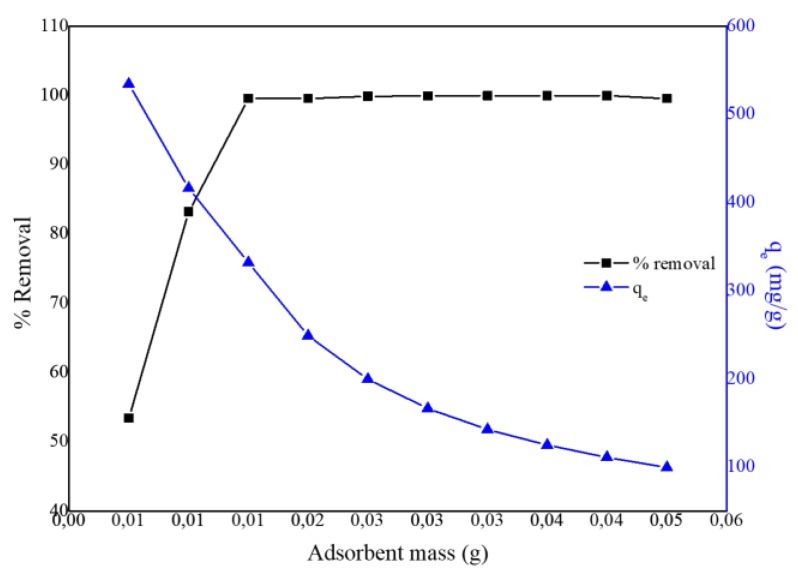
Effect of PPy-*m*PD/Fe_3_O_4_ adsorbent dose on the removal of Cr(VI) from the solution.

The effect of adsorbent dosage was performed to determine the minimum amount of PPy–*m*PD/Fe_3_O_4_ nanocomposite required to remove (% removal) and the adsorption equilibrium (*q*_e_) of the Cr(VI) from the solution at pH 2 (100 mg/L, 50 mL). The % removal and *q*_e_ results are presented in Figure 6. By varying the adsorbent dosage (0.005–0.05 g), the minimum amount of nanocomposite required to remove Cr(VI) from aqueous solution entirely was found to be 0.025 g.

**Figure 7:**
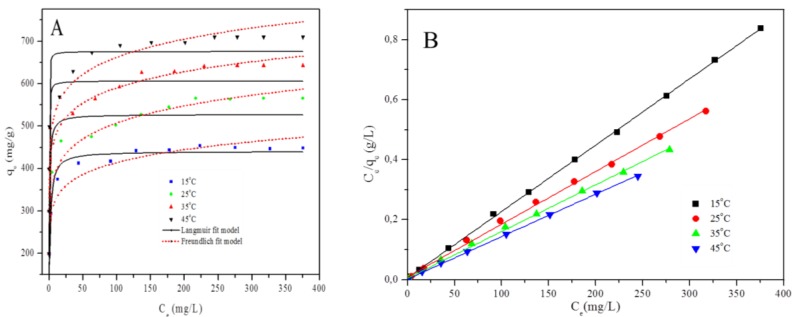
Isotherm data to the Langmuir and Freundlich models (**A)** and Isotherm data to the linear form of the Langmuir model for Cr(VI) adsorption (**B)** by PPy-*m*PD/Fe_3_O_4_ nanocomposite adsorbent.

Adsorption isotherms are pivotal in studying the interaction between the adsorbent and the adsorbate, as well as to evaluate the maximum capacity of the adsorbent [23]. The temperature effect on Cr(VI) uptake by the PPy–*m*PD/Fe_3_O_4_ nanocomposite was conducted at various temperatures (15–45 °C). The adsorption isotherms in Figure 5a show that the adsorption process is temperature-dependent (i.e., endothermic). This behaviour is supported by an increase in equilibrium adsorption capacity with increasing temperature. The obtained adsorption isotherm data were fitted to two isotherm models (Langmuir and Freundlich). It is known that the Freundlich isotherm is a well-known empirical model [28,29,30], which is not restricted to the formation of a monolayer. Figure 7a,b show the plots obtained from fitting the data to different forms of the isotherm models, and the calculated isotherm parameters are presented in Table 1. The correlation coefficients (*R*^2^) obtained for the linear and nonlinear Langmuir model are 0.99 and 0.95, respectively. In the case of the Freundlich model, *R*^2^ = 0.93 and 0.87 for linear (Figure S3) and nonlinear (Figure 7a) forms, respectively. From the table, it can be observed that the data fitted the Langmuir model better (Figure 7b) than the Freundlich model (Supporting Information). This implies that the surface of the nano-adsorbent is homogeneous (without the inter-reaction amongst adsorbed species), and with all the active adsorption sites equal in energy [2]. Furthermore, the monolayer surface coverage assumed by the Langmuir model can be associated with the electrostatic interaction occurring amongst Cr(VI) ions and the protonated PPy-*m*PD/Fe_3_O_4_ [30].

Another important parameter related to the Langmuir model is the dimensionless constant *R_L_*, which is used to determine whether the adsorption process is favourable or unfavourable. This separation factor was obtained from Equation (15):

(15) $$R_{L}=\frac{1}{1+K_{L}C_{0}}$$

The *R_L_* value indicates favourable adsorption for 0 < *R_L_* < 1. The *R_L_* values in Table 1 are between 0 and 1, and this is an indicative of a favourable adsorption process of Cr(VI). The maximum adsorption capacity of the PPy–*m*PD/Fe_3_O_4_ adsorbent for Cr(VI) removal was 555.6 mg/g at 25 °C. This value was higher than the maximum adsorption capacities reported for other magnetic composites (Table S1) [2,3,9,16,17,18,23,31,32,33,34,35,36,37,38]. Therefore, the incorporation of Fe_3_O_4_ nanoparticles into PPy–*m*PD (*q_m_* = 526.3 mg/g) did not only have an influence on the magnetic properties, but also increased the maximum adsorption capacity, both of which are preferable for water treatment (as the recovery of the material from treated water will be easier owing to the high magnetic property of the nanocomposite) [3]. Generally, the adsorption capacity increased with an increase in initial metal ions concentration present in the solution [33,34]. This was supported by the thermodynamic properties of the synthesised PPy, the magnetite nanoparticles and the PPy–*m*PD/Fe_3_O_4_ nanocomposite for Cr(VI) adsorption.

Table S2 shows the summary of Δ*G*^0^, Δ*H*^0^ and Δ*S*^0^ change values obtained at 15, 25, 35 and 45 °C. The negative values of Δ*G*^0^ suggest that Cr(VI) adsorption occurs spontaneously at all of the temperatures studied. The negative change in Δ*G*^0^ becomes more negative with increasing temperature for Cr(VI) adsorption by the PPy-*m*PD/Fe_3_O_4_ nanocomposite, implying the feasibility and spontaneous nature of the adsorption process. The slope and the intercept were used to determine the Δ*H*^0^ and Δ*S*^0^ of the adsorption. The positive value of Δ*H*^0^ confirmed that adsorption of Cr(VI) onto PPy-*m*PD/Fe_3_O_4_ nanocomposite was endothermic [35,39], while the positive value of Δ*S°* indicates that there is a high affinity between Cr(VI) and PPy-*m*PD/Fe_3_O_4_ nanocomposite.

**Figure 8:**
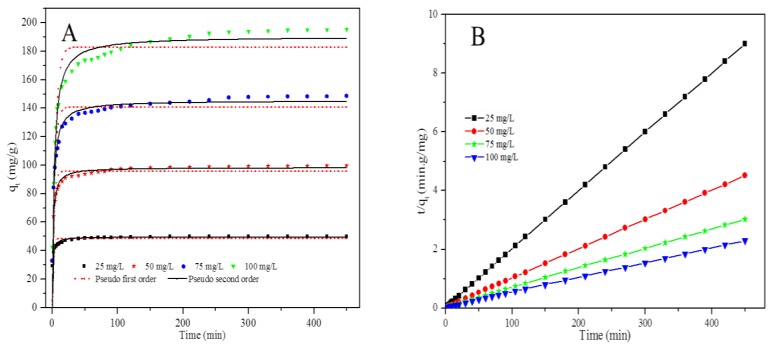
Nonlinear fitting of the data to the pseudo-first-order (PFO) and pseudo-second-order (PSO) kinetic models (**A**) and Fit of the data to the linear form of the PSO kinetic model (**B**) for Cr(VI) adsorption by the PPy–*m*PD/Fe_3_O_4_ nanocomposite.

The effect of contact time on Cr(VI) removal by the PPy–*m*PD/Fe_3_O_4_ nanocomposite adsorbent was investigated with four various Cr(VI) initial concentrations, i.e., 25, 50, 75 and 100 mg/L, and the results are depicted in Figure 8 and Figure S4. The figure demonstrates an increase in Cr(VI) uptake with an increase initial Cr(VI) concentrations. The observed increase in Cr(VI) removal with an increase in concentration is due to a large number of Cr(VI) species available in solution, resulting in more collisions between Cr(VI) ions and the nanocomposite surface in the solution [6].

Figure 8a shows that the kinetics data fitted the nonlinear PSO kinetic model better than the nonlinear PFO kinetic model. Figure 8b and Figure S4 shows the plots obtained from fitting of the kinetics data to the linear forms of PFO and PSO kinetic models, respectively.

Table S3 shows adsorption kinetic parameters obtained from the linear and nonlinear fitting of the data to the PFO and PSO models. The *R^2^* values obtained for the linear and nonlinear fittings of the data to the PFO model are lower than those obtained for the PSO kinetic model, suggesting that the adsorption of Cr(VI) onto PPy–*m*PD/Fe_3_O_4_ nanocomposite followed PSO kinetics [25]. Additionally, the obtained *q_e_* values for the PSO model were comparable to the *q_e_* values obtained experimentally. The PSOR constants (*k*_2_) values decreased with increasing initial Cr(VI) concentration, showing that the rate of Cr(VI) adsorption by the PPy–mPD/Fe_3_O_4_ nanocomposite increased as the Cr(VI) initial concentration decreased [7]. Adsorption kinetics data were used to determine the rate-limiting step (RLS) of Cr(VI) adsorption by the nanocomposite, by fitting the data to the Weber and Morris intra-particle diffusion model (Figure S5). Figure S5 and Table S4 show the *k_i_* and *C* values for three different linear segments obtained for each plot. The results indicate that the three stages control the adsorption process, and are rate limiting at a certain range in time. Furthermore, the larger values of *k_i_* suggest that film diffusion is faster, and has a more significant role as the RLS [34,36,37,38]. In addition, temperature-dependent kinetics data were used to calculate the activation energy (*E_a_*) for Cr(VI) adsorption by the PPy–*m*PD/Fe_3_O_4_ nanocomposite obtained at 15, 25, 35 and 45 °C, and the results are provided in Table S2. The *E_a_* of the adsorption process can be related to the PSOR constant, *k_2_* (g/mg/min) by the linearised Arrhenius (lnk2 = lnA − $\frac{E_{a}}{RT}$), *T* is the temperature *T* (K), *A* (g/mg/min) is the frequency factor, *R* denotes the general gas constant (J/mol/K), and *E_a_* (kJ/mol) is the activation energy. The results show an increase in the rate of Cr(VI) adsorption with an increase in temperature, which gave an *E*_a_ = 0.0258 kJ/mol determined from the Arrhenius plot.

**Figure 9:**
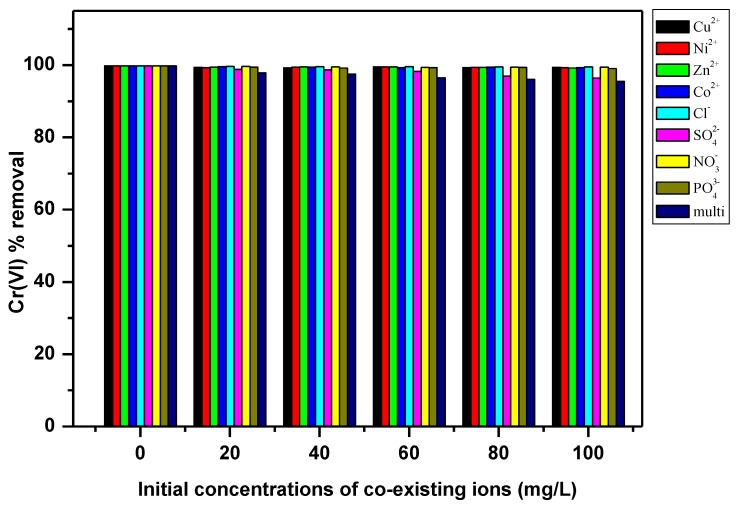
Effect of coexisting ions on Cr(VI) adsorption by the PPy–*m*PD/Fe_3_O_4_ nanocomposite.

The effect of selected ions (Cl^−^, Co^2+^, Cu^2+^, Ni^2+^, Zn^2+^, NO_3_^−^, PO_4_^3−^ and SO_4_^2−^) on the Cr(VI) removal by the PPy–*m*PD/Fe_3_O_4_ nanocomposite was studied by varying the coexisting ions concentrations in solutions from 20 to 100 mg/L, with the Cr(VI) concentration at x mg/L. Figure 9 revealed that the four cations (Cu^2+^, Co^2+^, Zn^2+^, Ni^2+^) had no significant effect on the removal of Cr(VI). This observation was anticipated, since the adsorption of Cr(VI) at pH 2 takes place via anion exchange with dopant Cl^−^ ions in the polymer, and consequently, the PPy-*m*PD/Fe_3_O_4_ nanocomposite has no affinity for cations [25,39]. Considering that Cr(VI) exists as an oxyanion, the presence of other anionic compounds may affect its adsorption onto the PPy-mPD/Fe_3_O_4_ nanocomposite. Hence, the effects of anions (Cl^−^, NO_3_^−^, PO_4_^3−^ and SO_4_^2−^) were examined to assess competition with Cr(VI) during adsorption. Figure 9 again showed that when the concentration of anions increases to 100 mg/L, there was a slight decrease of ≈3% in the removal of Cr(VI) by PPy-*m*PD/Fe_3_O_4_ nanocomposite, due the SO_4_^2−^ ions. Even though high concentration of SO_4_^2−^ existed, the removal of Cr(VI) by the PPy-*m*PD/Fe_3_O_4_ nanocomposite was still not hindered significantly. The insignificant effect of low affinity ligands Cl^−^ and NO_3_^−^ on Cr(VI) removal by the PPy-*m*PD/Fe_3_O_4_ nanocomposite may be because these ligands only form weak outer sphere complexes with binding sites on the adsorbents [26]. SO_4_^2−^ has the ability to form both inner and outer sphere complexes, but as a result of its high hydration energy, as compared to that of Cr(VI), and therefore does not affect the removal of Cr(VI) by the PPy-*m*PD/Fe_3_O_4_ nanocomposite [17,18]. The high selective behaviour of the PPy-*m*PD/Fe_3_O_4_ nanocomposite towards Cr(VI) may be attributed to the ability of PPy to reduce toxic Cr(VI) to less toxic Cr(III), because of a high positive redox potential of HCrO_4_^−^ anions, the main form of Cr(VI) in solutions at low pH [3].

**Figure 10:**
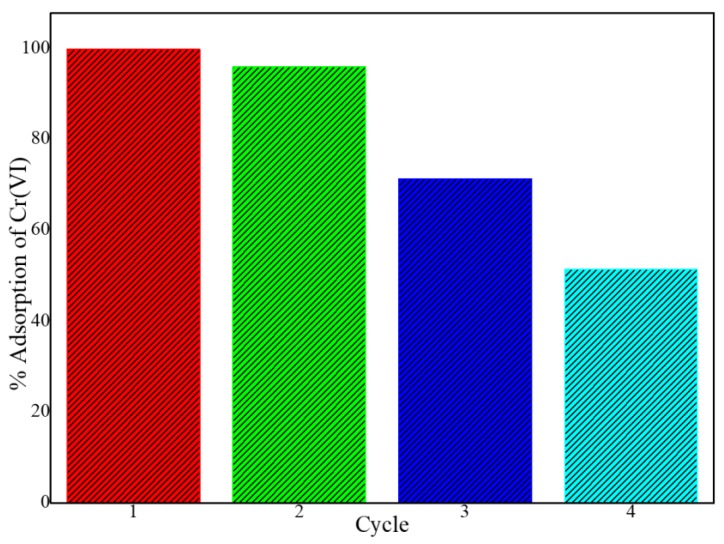
Adsorption cycles for Cr(VI) removal by the PPy–*m*PD/Fe_3_O_4_ nanocomposite.

The regeneration and reusability of adsorbents are important aspects to consider for the economic sustainability of water purification processes. The potential to reuse the PPy-*m*PD/Fe_3_O_4_ nanocomposite was assessed by conducting four consecutive adsorption–desorption–regeneration cycles. For the desorption of Cr(VI) from the nanocomposite, an NH_4_OH solution of 0.1 M was used, followed by regeneration using 2 M HCl solution before being used for the next cycle. The results in Figure 10 showed that the PPy-*m*PD/Fe_3_O_4_ nanocomposite could be used for two consecutive cycles without a significant reduction in the adsorption capacity, as the removal efficiency was still above 90% in the second cycle. In the third and the fourth cycles, 70% and 50% Cr(VI) removal efficiency by the PPy-*m*PD/Fe_3_O_4_ nanocomposite was obtained, respectively. This may be attributed to an overoxidation of the PPy-*m*PD/Fe_3_O_4_ nanocomposite, which caused the deterioration of the polymer, and therefore reduced the adsorption sites available for Cr(VI) [26]. From these findings, it must be stated that this is only preliminary desorption research, and they should be continued to find a better desorption–regeneration procedure to prove the sorbent to be useful in practice.

## 4. Conclusions

A novel magnetic PPy–*m*PD/Fe_3_O_4_ nanocomposite with a saturation magnetisation of 6.6 emu/g was synthesised for the removal of Cr(VI) from aqueous solution. The adsorption isotherms were obtained at temperatures (15–45 °C) and varying the Cr(VI) initial concentration (100–600 mg/L). The adsorption isotherm data obeyed the Langmuir isotherm model, and a higher Langmuir maximum adsorption capacity of 555.6 mg/g was obtained for Cr(VI) removal by the adsorbent at pH 2 and 25 °C. The PPy–*m*PD/Fe_3_O_4_ nanocomposite showed high selectivity towards Cr(VI) adsorption, since the presence of competing ions in solution had no significant effect. Based on the previous results, it can be concluded that the ability to synthesise a magnetic PPy–*m*PD/Fe_3_O_4_ nanocomposite containing an increased number of amine functional groups as a very low cost and super-adsorbent material for heavy metal ions was effectively achieved. Thus, due to the high adsorption capacity and selectivity for Cr(VI), the PPy–*m*PD/Fe_3_O_4_ nanocomposite is a potential adsorbent for Cr(VI) removal from industrial wastewater.

## Figures and Tables

**Table 1:** Isotherm parameters for Cr(VI) adsorption by PPy–*m*PD/Fe_3_O_4_ using both Langmuir and Freundlich models.

Isotherm Model	Temperature			
	15 °C	25 °C	35 °C	45 °C
Langmuir	-			
*Linear*				
*q_m_*	454.5	555.6	625	714.3
*K_L_*	0.338	0.194	0.314	0.583
*R_L_*	0.00838	0.01451	0.00902	0.00488
*R* ^2^	0.9997	0.9982	0.9986	0.9996
*Non-linear*	-			
Best-fit values				
*q_m_*	440.6	522.4	596.5	661.0
*K_L_*	0.7719	1.914	8.046	13.05
Std. Error	-			
*q_m_*	6.038	15.80	15.01	19.06
*K_L_*	0.09790	0.5212	1.483	2.537
95% Confidence Intervals	-			
*q_m_*	426.9–454.2	486.6–558.1	562.6–630.5	617.9–704.1
*K_L_*	0.5505–0.9934	0.7353–3.093	4.692–11.40	7.317–18.79
Goodness of Fit	-			
Degrees of Freedom	9	9	9	9
*R* ^2^	0.9611	0.9536	0.9310	0.9250
Absolute Sum of Squares	2592	6727	15505	24061
Sy.x	16.97	27.34	41.51	51.71
Number of points	-			
Analysed	11	11	11	11
Freundlich	-			
*Linear*				
*K_F_*	240.495	287.349	370.369	415.715
*N*	8.46	7.94	9.5	9.18
*R* ^2^	0.8829	0.9358	0.8536	0.845
*Non-linear*	-			
Best-fit values				
*K_F_*	259.1	303.1	388.2	435.5
*N*	9.816	8.847	10.76	10.47
Std. Error	-			
*K_F_*	17.73	14.53	21.38	23.13
*N*	1.342	0.7892	1.391	1.319
95% Confidence Intervals	-			
*K_F_*	219.0–299.2	270.3–336.0	339.8–436.6	383.2–487.8
*N*	6.780–12.85	7.062–10.63	7.613–13.91	7.482–13.45
Goodness of Fit	-			
Degrees of Freedom	9	9	9	9
*R* ^2^	0.8899	0.8747	0.9052	0.9102
Absolute Sum of Squares	7329	18170	21322	28790
Sy.x	28.54	44.39	48.67	56.56
Number of points	-			
Analysed	11	11	11	11

Units: *q_m_*: mg/g, *K_L_*: L/mg, *K_F_*: mg/g.

## Supplementary Materials

The following are available online at https://www.mdpi.com/2073-4360/12/3/679/s1, Figure S1: Plot to obtain the thermodynamic parameters for Cr(VI) adsorption by the PPy–*m*PD/Fe_3_O_4_ nanocomposite, Figure S2: The point of the zero charge of the PPy-*m*PD/Fe_3_O_4_ nanocomposite, Figure S3: Linear fit of temperature effect data to Freundlich isotherm model for the PPy-*m*PD/Fe_3_O_4_ nanocomposite, Figure S4: Linear fit of pseudo second order kinetic model for Cr(VI) adsorption by the PPy-*m*PD/Fe_3_O_4_ nanocomposite, Figure S5: Plots obtained for the intra-particle diffusion (IPD) model for Cr(VI) adsorption by the PPy–*m*PD/Fe_3_O_4_ nanocomposite, Table S1: Adsorption capacity of the PPy–*m*PD/Fe_3_O_4_ nanocomposite adsorbent, studied in comparison to other adsorbents reported in the literature for the removal of Cr(VI) from solution, Table S2: Thermodynamic values for Cr(VI) adsorption by the PPy–*m*PD/Fe_3_O_4_ nanocomposite, Table S3: Kinetics parameters of Cr(VI) adsorption by the PPy–*m*PD/Fe_3_O_4_ nanocomposite, Table S4: Intra-particle diffusion model parameters of Cr(VI) adsorption by the PPy–*m*PD/Fe_3_O_4_ nanocomposite.
