# Supplementary material for: Influence of Magnetic Nanoparticles on Modified Polypyrrole/m-Phenylediamine for Adsorption of Cr(VI) from Aqueous Solution
Source: Polymers (Basel). 2020 Mar 19;12(3):679. doi: 10.3390/polym12030679 (PMC7182836; doi:10.3390/polym12030679)
Supplement: Supplementary file 1 [file polymers-12-00679-s001.pdf]

Influence of Magnetic Nanoparticles on Modified Polypyrrole/*m*-Phenylediamine for Adsorption of Cr(VI) from Aqueous Solution

Thabiso Carol Maponya, Kabelo Edmond Ramohlola, Nazia Hassan Kera, Kwena Desmond Modibane, Arjun Maity, Lebogang Maureen Katata-Seru and Mpitloane Joseph Hato

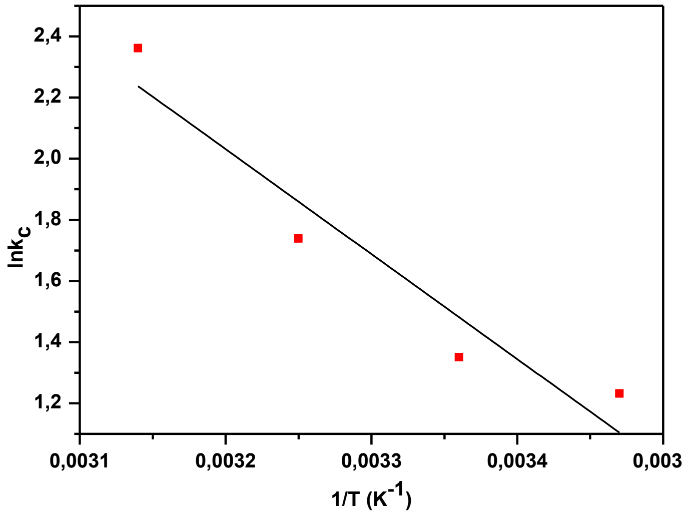

Figure S1. Plot to obtain the thermodynamic parameters for Cr(VI) adsorption by the PPy–*m*PD/Fe_3O_4 nanocomposite.

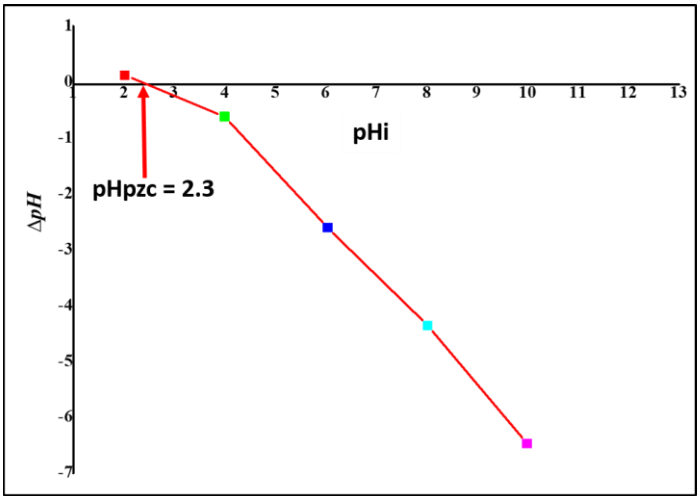

Figure S2. The point of the zero charge of the PPy-*m*PD/Fe_3O_4 nanocomposite.

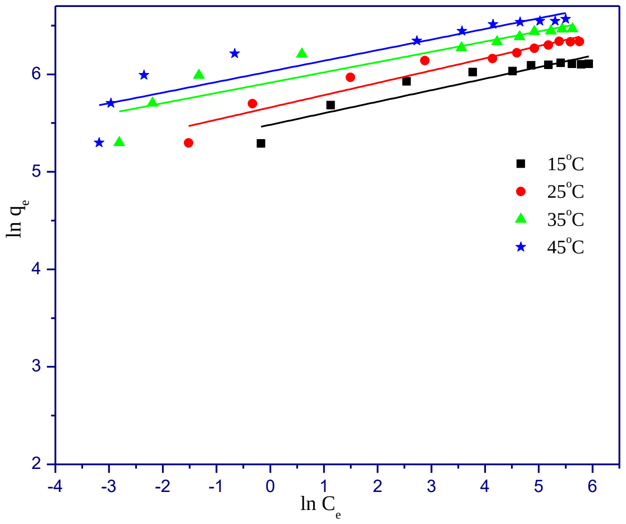

Figure S3. Linear fit of temperature effect data to Freundlich isotherm model for the PPy-*m*PD/Fe_3O_4 nanocomposite.

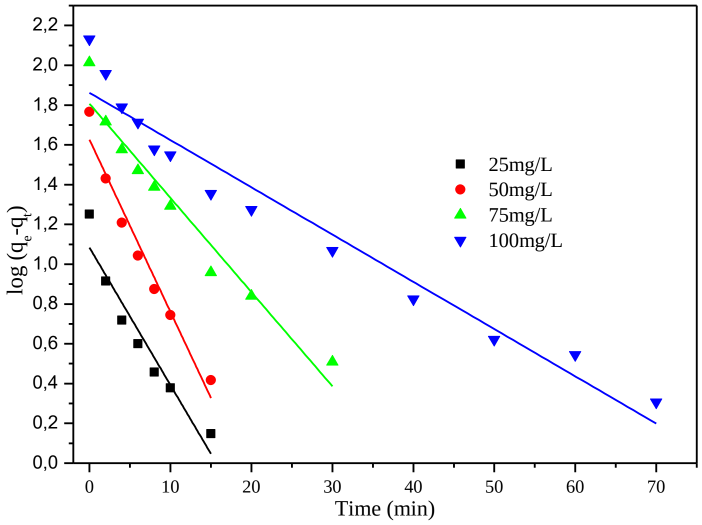

Figure S4. Linear fit of pseudo second order kinetic model for Cr(VI) adsorption by the PPy-*m*PD/Fe_3O_4 nanocomposite.

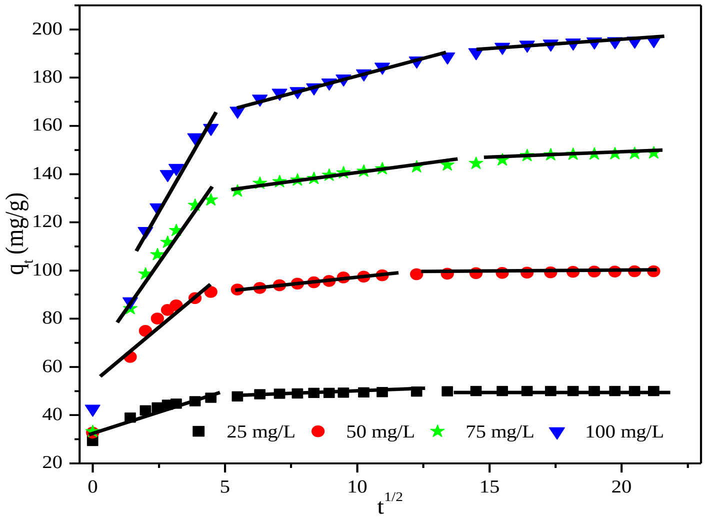

Figure S5. Plots obtained for the intra-particle diffusion (IPD) model for Cr(VI) adsorption by the PPy–*m*PD/Fe_3O_4 nanocomposite.

Table S1. Adsorption capacity of the PPy–*m*PD/Fe_3O_4 nanocomposite adsorbent, studied in comparison to other adsorbents reported in the literature for the removal of Cr(VI) from solution.

Adsorbents	Qmax (mg/g)	pH	Adsorbent Dose (mg)	Initial Cr(VI) Concentration (mg/L)	References
Magnetic-MWCNT	14.28	5	5	11.5	[1]
Magnetic-Activated Carbon	2.8	5	5	11.5	[1]
Fe_3O_4/CTAB	18.5	4	12	100	[2]
Fe_3O_4-Graphene Oxide	31.8	3	50	50	[3]
Fe_3O_4-GS	17.29	1- 3.5	8 or 10	3.0	[4]
PmPD/rGO/NFO	502.5	3	10	50	[5]
PPy-*m*PD/Fe_3O_4	555.6	2	20	100	Present study

Cetyletrimethylammonium bromide (CTAB), multi-walled carbon nanotube (MWCNT), graphenes magnetic material (Fe_3O_4-GS), poly(m-phenylenediamine)/reduced graphene oxide/nickel ferrite (PmPD/rGO/NFO)

Table S2. Thermodynamic values for Cr(VI) adsorption by the PPy–*m*PD/Fe_3O_4 nanocomposite.

Temperature (°C)	ΔG^0 (kJ/mol)	ΔH^0 (kJ/mol)	ΔS^0 (kJ/K mol)
15	−2.949	28.55	0.108
25	−3.347	-	
35	−4.452	-	
45	−6.244	-	

Table S3. Kinetics parameters of Cr(VI) adsorption by the PPy–*m*PD/Fe_3O_4 nanocomposite.

Kinetic model	Initial Cr(VI) concentration Concentration			
	25 mg/L	50 mg/L	75 mg/L	100 mg/L

**Pseudo-first order (PFO)**	-			
Linear				
q_e *(exp)*	47.160	91.054	136.148	177.921
k_1	0.159	0.199	0.109	0.055
R^2	0.9281	0.9661	0.9515	0.9538
Non-linear Best-fit values	-			
q_e	48.62	95.84	140.9	183.2
k_1	0.6684	0.4029	0.2757	0.2031
Std. Error	-			
q_e	1.227	1.643	2.287	3.201
k_1	0.2143	0.06375	0.03407	0.02393
95 % Confidence Intervals	-			
q_e	46.10–51.14	92.46–99.22	136.2–145.6	176.6–189.8
k_1	0.2277–1.109	0.2718–0.5340	0.2056–0.3457	0.1540–0.2523
Goodness of Fit	-			
Degrees of Freedom	26	26	26	26
R^2	0.7143	0.7054	0.8272	0.8433
Absolute Sum of Squares	963.4	1628	3002	5654
Sy.x	6.087	7.912	10.74	14.75
Number of points	-			
Analysed	28	28	28	28
**Pseudo-second order (PSO)**	-			
Linear				
q_e	50.251	100	149,25	196.08
k_2	0.0172	0.004386	0.001694	0.0009289
R^2	1	1	0.9998	0.9997
Non-linear Best-fit values	-			
q_e	49.60	98.32	145.3	190.3
k_2	0.02859	0.008034	0.003512	0.001784
Std. Error	-			
q_e	1.312	1.541	1.792	2.510
k_2	0.01419	0.001689	0.000474	0.0002209
95 % Confidence Intervals	-			
q_e	46.90–52.30	95.15–101.5	141.6–148.9	185.2–195.5
k_2	0.000583–0.05776	0.004562–0.01151	0.002536–0.004489	0.001329–0.002238
Goodness of Fit	-			
Degrees of Freedom	26	26	26	26
R^2	0.5566	0.7941	0.9169	0.9273
Absolute Sum of Squares	874.7	1138	1143	2621
Sy.x	5.800	6.615	7.449	10.04
Number of points	-			
Analysed	28	28	28	28

Units: q_e: mg/g, k_1: 1/min, k_2: g/mg*min.

Adsorption kinetics data were used to determine the rate-limiting step (RLS) of Cr(VI) adsorption by the nanocomposite, by fitting the data to the Weber and Morris intra-particle diffusion model represented by the following expression:

$$q_t = k_i t^{0.5} + C \quad \text{(S1)}$$

where k_i represents the rate constant measured in (mg/g.min$^{0.5}$), and C (mg/g) denotes the intercept associated with the boundary layer thickness.

Table S4. Intra-particle diffusion model parameters of Cr(VI) adsorption by the PPy–*m*PD/Fe_3O_4 nanocomposite.

Initial Concentration (mg/L)	Regions	Parameters		
		k_i (mg/g/min$^{0.5}$)	C	R^2
25	1st	3.1359	33.607	0.8364
	2nd	0.1783	47.568	0.9699
	3rd	0.0159	49.682	0.8086
50	1st	12.673	43.025	0.8785
	2nd	1.1275	85.804	0.9851
	3rd	0.1463	96.671	0.9771
75	1st	23.92	43.07	0.9461
	2nd	1.6608	124.13	0.9295
	3rd	0.6408	135.91	0.8406
100	1st	33.116	43.709	0.9867
	2nd	3.8529	143.63	0.9530
	3rd	0.9212	177.17	0.8835

References:

1. Bhaumik, M.; Maity, A.; Srinivasu, V.V.; Onyango, M.S. Removal of hexavalent chromium from aqueous solution using polypyrrole-polyaniline nanofibers. *Chem. Eng. J.* **2012**, *181*, 323–333.
2. Salam, M.A. Preparation and characterization of chitin/magnetite/multiwalled carbon nanotubes magnetic nanocomposite for toxic hexavalent chromium removal from solution. *J. Mol. Liq*. **2017**, *233*, 197–202.
3. Bhaumik, M.; McCrindle, R.; Maity, A. Efficient removal of Congo red from aqueous solutions by adsorption onto interconnected polypyrrole-polyaniline nanofibers. *Chem. Eng. J.* **2013**, *228*, 506–515.
4. Guo, X.; Du, B.; Wei, Q.; Yang, J.; Hu, L.; Yan, L.; Xu, W. Synthesis of amino functionalized magnetic graphenes composite material and its application to remove Cr(VI), Pb(II), Hg(II), Cd(II) and Ni(II) from contaminated water. *J. Hazard. Mater*. **2014**, *278*, 211–220.
5. Wang, W.; Cai, K.; Wu, X.; Shao, X.; Yang, X. A novel poly(m-phenylenediamine)/reduced graphene oxide/nickel ferrite magnetic adsorbent with excellent removal ability of dyes and Cr(VI). *J. Alloys Compd.* **2017**, *722*, 532–543.
